# Endocrinal metabolic regulation on the skeletal system in post-menopausal women

**DOI:** 10.3389/fphys.2022.1052429

**Published:** 2022-11-11

**Authors:** Santosh Thapa, Ananya Nandy, Elizabeth Rendina-Ruedy

**Affiliations:** ^1^ Department of Medicine, Division of Clinical Pharmacology, Vanderbilt University Medical Center, Nashville, TN, United States; ^2^ Molecular Physiology and Biophysics, Vanderbilt University, Nashville, TN, United States

**Keywords:** osteoporosis, osteoblasts, post-menopause, estrogen, bone cell metabolism

## Abstract

Osteoporosis is a common endocrinologic disorder characterized as a chronic bone loss condition. Sexual dimorphism is ubiquitous in the incidence of osteoporosis with post-menopausal women being acutely affected. Gonadal sex hormones including estrogen act as crucial regulators of bone mass; therefore, loss of such hormones leads to an imbalance in skeletal turnover leading to osteoporosis. Estrogen can influence both bone formation as well as resorption by reducing osteoblast activity and enhancing osteoclastogenesis. Additionally, estrogen is a potent regulator of systemic metabolism. Recent studies have provided clues that estrogenic effect on bone might also involve alterations in bone cell metabolism and bioenergetic potential. While direct effects of gonadal hormones ability to alter intracellular metabolism of bone cells has not been studied, there is precedence within the literature that this is occurring and contributing to post-menopausal bone loss. This review aims to serve as a perspective piece detailing the prospective role of gonadal hormones regulating bone cell metabolic potential.

## Graphical Abstract

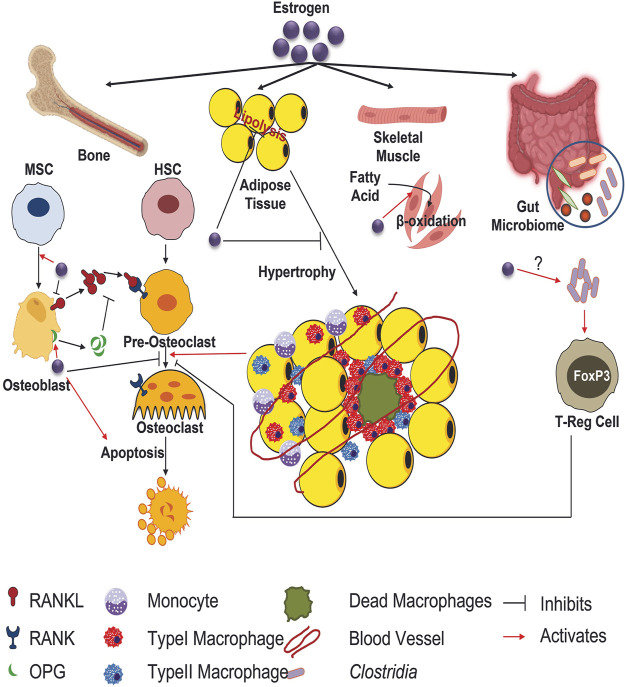

## 1 Skeletal health and osteoporosis prevalence

The global trend of increased life expectancy has no sign of abating and as a consequence, the demographic continues to shift towards an aged population at an unprecedented rate. With advancing age, it is noted that an overwhelming number of populations will develop age related degenerative musculoskeletal disorder such as loss of bone mass or osteoporosis contributing to increased morbidity and substantial long-term loss of independence (Kanis et al., 2008). Osteoporosis is characterized by chronic low bone mineral density (BMD) and is a global public health concern posing a serious socio-economic and health burden (Kanis et al., 2008). While low BMD itself is not known to pose a health threat, this decrease in BMD is often associated with bone fragility leading to increased non-traumatic fracture susceptibility with fracture being a major clinical event (Clynes et al., 2020). Major osteoporotic fractures primarily occur in three regions: hip, vertebral and forearm (radius/ulna) (Lad et al., 2007). A report from Johnell and Kanis concluded that worldwide a staggering nine million osteoporotic fractures occur annually, this equates to an osteoporotic fracture every 3 seconds (Johnell and Kanis, 2006). Among these sites, hip fracture is considered as the most acute form of osteoporotic fracture associated with increased mortality, debility, and destitution (Cooper et al., 2011; Tajeu et al., 2013).

Bone loss associated with aging can be contributed by multiple extrinsic factors like decreased physical activity, nutritional deficiency, consumption of alcohol and tobacco (Marie and Kassem, 2011) as well as by intrinsic factors at the molecular level like stem cell atrophy, decreased proliferation, differentiation and life span of osteoblasts, accumulation of oxidative damage and cell death etc., (Kassem and Marie, 2011). Arguably, one of the most noteworthy life events that occurs with aging and coincides with decline in BMD relates to gonadal or sex hormones. While this affects both men and women, the dramatic and sudden loss of estrogen in women that occurs during menopause is a potent etiological factor that leads to an imbalance in skeletal turnover resulting in osteoporosis (Ji and Yu, 2015). As such, it is generally accepted that menopause decreases bone forming osteoblasts along with osteoblastic-lineage cells such as osteocytes, while increasing bone resorbing osteoclasts. These alterations in cellular populations and function are therefore reflected in net loss of bone density. In this capacity, we have been struck by the metabolic alterations estrogens typically exert on various tissues, with little to no mention of how bone cells metabolism is altered during menopause. Moreover, as the field of bone cell bioenergetics expands, this review serves as a perspective piece connecting metabolic alterations occurring in during menopause and how these changes can both directly and indirectly impact skeletal homeostasis.

In general, bone strength is determined not merely by its materialistic characteristics (e.g., mineral density), rather by the macroscopic (shape, dimension) and microscopic (osteons orientation) geometry within the bone tissue (Hart et al., 2020). Osteoporosis is further subdivided into primary osteoporosis and secondary osteoporosis. The primary osteoporosis includes postmenopausal osteoporosis (Type I), caused due to the estrogen deficiency, and senile osteoporosis (Type II), whereas the secondary osteoporosis encompasses etiological mechanism (malabsorption, glucocorticoids, hyperparathyroidism) (Mirza and Canalis, 2015).

## 2 Primary osteoporosis–type I/post-menopause and type II/Age)

Menopause occurs in women on average at an age of 51 and is defined as 12 concurrent months without menses. In its entirety, menopause is described as having four distinct stages: perimenopause, early menopause, menopause, and post-menopause. Estrogen levels begin to decline during the peri-menopausal stage as gradual ovarian failure occurs, with full estrogen decline during menopause and post-menopausal stages. As a compensatory mechanism, follicle stimulating hormone (FSH) increases during menopause and remains elevated post-menopause. Estrogen, and to some extent FSH, are critical regulators of bone turnover. Therefore, skeletal health is directly impacted during all the stages of menopause, with bone loss occurring primarily during the menopause and post-menopausal stage (Ji and Yu, 2015).

### 2.1 Clinical detection of bone loss during menopause

The diagnosis of osteoporosis is based on the assessment of BMD and bone fragility. There are diverse methods which are used to measure these parameters. These include but not limited to dual energy x-ray absorptiometry (DXA), single photon absorptiometry, quantitative computed tomography (QCT), quantitative ultrasound (QUS), digital X-ray radiogrammetry, radiographic absorptiometry, ultrasonography (US) and magnetic resonance (MR) (Kanis et al., 2019; D'Elia et al., 2009; Ahlborg et al., 2003). Among them, DXA is the most versatile and ubiquitously exploited bone densitometric technique making it the “gold standard” for diagnosing osteoporosis. Overall, studies have shown substantial variation in osteoporotic related hip fracture incidence rates around the globe. The removal of estrogen in menopausal women has wider effects than in men of similar age group. One out of three women over the age of 50 will experience osteoporosis related bone fracture (Shuhart et al., 2019).

### 2.2 Molecular mechanism of gonadal hormones role in bone turnover

Bone undergoes continuous remodeling whereby old bone is broken down and removed by the osteoclast for subsequent bone to be deposited by the osteoblast. Gonadal hormones such as estrogen has been shown to have a profound impact on the bone remodeling process (Almeida et al., 2017). As one might predict, given the sharp decrease in BMD during menopause, loss of estrogen increases osteoclastogenesis and activity of osteoclasts thereby accelerating bone resorption (Moller et al., 2020). This level of increased skeletal resorption is unable to be restored by the osteoblast as osteoblastogenesis and bone formation are generally reduced, thus leading to the loss of bone density and increase in fracture in post-menopausal women. Single photon absorptiometry study done in 108 women over a period of 15 years from the time of menopause has shown an annual decrease in bone mineral density and increase in periosteal diameter. The post-menopausal estradiol level is correlated with changes in both bone mineral density and periosteal diameter (Ahlborg et al., 2003).

Several lines of evidence have lent credence that estrogen has profound impact on multiple cells in the skeletal niche (Seko et al., 2020). First, estrogen contributes to bone formation by extending osteogenic differentiation of mesenchymal stem cells (MSCs) and osteoblast maturation as well as inducing osteoclast apoptosis. For example, estrogen promotes the osteoblastic production of insulin-like growth factor (IGF)-1 and IGF- β as well as pro-collagen synthesis (Locatelli and Bianchi, 2014). There is a reduced periosteal cells proliferation and differentiation in ERα deleted mice in mesenchymal progenitors. This is due to the decrease in canonical Wnt signaling triggering low cortical bone mass (Melville et al., 2014). Notably, Wnt pathway is imperative for the maturation and formation of osteoblast as well as bone mass accrual. Terminal differentiated osteocytes express sclerostin (*Sost*) which has also been shown to be influenced by sex hormones. For example, postmenopausal women tend to demonstrate higher levels of *Sost* compared with premenopausal women, however, estradiol treatment significantly lowered *Sost* (Mödder et al., 2011). In addition to cells of the osteoblastic lineage, estrogen also plays a central role in osteoclast biology. Molecularaly, estrogen regulates the bone metabolism *via* two receptors namely, 1) estrogen receptor-alpha (ERα) and beta (ERβ), with ERα being more dominant (Sims et al., 2003). In addition to the receptor activator of nuclear factor-_k_B ligand (RANKL, also known as tumor necrosis factor ligand superfamily member 11) and osteoprotegerin (OPG) inflammatory cytokines (i.e., IL-1, IL-6, TNF-α AND αVβ3 integrin) also promote osteoclastogenesis (Azuma et al., 2000; Teitelbaum, 2000; Yokota et al., 2014). In ovariectomized (OVX) rodent models of post-menopausal osteoporosis, osteoblasts increase their expression *Rankl*, while osteoclasts express higher levels of *Rank*, thus increasing osteoclastic bone resorption (Yang et al., 2021). Additionally, a study performed by Li et al. (2009) using male orchiectomized (ORX) rats reported a significant increase of free soluble RANKL in bone marrow plasma (Li et al., 2009). These data demonstrate that loss of estrogen directly regulates both osteoblast, osteocyte and osteoclast activity, ultimately reducing BMD.

In additional to women, estrogen plays an important role in the maintenance of male skeletal system as well. In fact serum estradiol levels in elderly men are higher than in post-menopausal women (Labrie et al., 2009) Alterations in the circulating levels of both androgen and estradiol have been shown to be associated with low bone mass and impaired bone strength (Gennari et al., 2008; Vandenput and Ohlsson, 2009; Wu and Zhang, 2018). Mutations in ER-α in male causing resistance to estrogen leads to osteopenia with a decrease in trabecular and cortical BMD, increase in bone turn over markers and unfused epiphysis (Smith et al., 1994). Estrogen has two different sources in male, 15% of the hormone is originated in testes whereas 85% comes from the conversion by peripheral aromatization of the circulating androgen by the enzyme aromatase (Simpson, 2000; Gennari et al., 2004). Mutation in the aromatase known as aromatase deficiency syndrome where male patients have undetectable amount of estrogen with normal or elevated level of androgen has been shown to be associated with impaired skeletal maturation and bone metabolism in males (Bouillon et al., 2004; Vandenput and Ohlsson, 2009; Merlotti et al., 2011). Estrogen therapy in these patients have been shown to improve skeletal health (Bilezikian et al., 1998; Rochira et al., 2000)

## 3. Alterations in metabolism during menopause

The menopausal transition is associated with significant weight gain. Specifically, this is associated with a decrease in energy expenditure and increase abdominal adiposity, along with dyslipidemia to include elevated cholesterol and triglycerides. In this regard the Healthy Women’s Study demonstrated an average weight gain of ∼5.5 lbs over a 30-year period over the menopausal transition (Williams et al., 2019). Noteworthy, even when kilocalories are controlled, women, and OVX mice gain more weight compared to controls (Ding et al., 2017). An explanation to this is a loss of lean mass and decline in basal energy expenditure. If further caloric intake is not reduced and/or not change in physical activity, these changes lead to increased visceral adipose tissue and body fat redistribution. Molecularly, the impact estrogen has on metabolism has been more complicated. For example, despite the increase in adiposity associated with menopause, estrogen has been shown to reduce lipolysis in adipose depots while supporting adipocyte proliferation and differentiation (i.e., hyperplasia and hypertrophy) (Steiner and Berry, 2022). This mechanism appears to be ER-α mediated, whereas ER-β mechanism may have opposing effects. Despite this contradictory finding, estrogen has been demonstrated to enhance fatty acid utilization and oxidation in skeletal muscle (Morselli et al., 2018). This finding provides an additional explanation as to why loss of estrogen during menopause leads to attenuated fatty acid oxidation, lower muscle mass, and reduced net energy expenditure. Additionally, greater serum free fatty acids have been reported in postmenopausal women compared to postmenopausal women receiving estrogen therapy. Therefore, estrogen acts both directly and indirectly to regulate metabolic function.

### 3.1 Potential mechanisms of metabolic alterations in bone cells

As previously mentioned, loss of estrogen results in reduced osteoblast mediated bone formation and enhanced osteoclastic resorption (Emmanuelle et al., 2021). Given the recent expansion of interest in understanding how these cellular processes are regulated *via* metabolic flux and bioenergetic capacity, there are several lines of indirect evidence that postmenopausal osteoporosis is a product of dysregulated intracellular metabolism in bone cells. Indeed, targeting metabolic pathways in bone cells is an incredibly provocative tool that could be applied to combat post-menopausal osteoporosis. In fact, first-generation anti-resorptive bisphosphonates (i.e., etidronate and clodronate) used to treat osteoporosis inhibit cellular energy (adenosine triphosphate or ATP) of the osteoclast (Drake et al., 2008; Malwal et al., 2018). While the N-containing second generation bisphosphonates target the cholesterol biosynthetic pathway. These widely prescribed drugs represent an impeccable example of how metabolic pathways can be exploited to impact overall bone health and improve patient quality of life.

#### 3.1.1 Glucose metabolism

Osteoblast metabolism has been generally well characterized by our lab and others. This includes the finding that bone formation by the osteoblast is viewed as an energy demanding process whereby extracellular matrix proteins are synthesized and secreted along with mineralization vesicles (Borle et al., 1960a; Borle et al., 1960b). Metabolic substrates capable of generating adenosine triphosphate (ATP), osteoblasts can utilize glucose, glutamine, and fatty acids (Karner et al., 2015; Wei et al., 2015; Kim et al., 2017). The ability for osteoblasts to utilize glucose as a substrate is widely accepted in the bone biology field (Esen et al., 2013; Wei et al., 2015). Relative to these findings, Wei et al. (2015) published data describing GLUT1’s (*Slc2a1*) as the primary glucose transporter on osteoblasts, whereas data exists that both GLUT3 and GLUT4 are also expressed (Thomas et al., 1996; Zoch et al., 2016). To this end, mature osteoblasts generate ATP *via* glucose substrates using aerobic glycolysis (Jayapalan et al., 2022). In fact, cellular metabolic programming has been suggested to be the determining factor in deciding the lineage fate of the stromal cells in mice (Tencerova et al., 2019a). Adipo-progenitor cells have been shown to be more responsive to insulin and dependent on oxidative phosphorylation whereas the osteo-progenitor cells are more glycolytic. Interestingly, up-regulating glycolysis in adipo-progenitor cells by treating them with parathyroid hormone (PTH) decreases their adipogenic potential (Tencerova et al., 2019a). Even mesenchymal stromal cells from obese human have been shown to exhibit higher oxidative phosphorylation tendency and higher adipogenic potential (Tencerova et al., 2019b). While it has yet to be studied directly, it is plausible estrogen, or lack thereof in menopause, could regulate osteoblast glucose metabolism. For example, estrogen has been shown to activate GLUT4 in the intestine and GLUT1 in the brain (Gregorio et al., 2021). Estrogens have also been demonstrated to enhance glycolysis *via* intermediate metabolite regulation of phosphofructokinase (Alemany, 2021). Taken together, it is possible that similar pathways are also altered in osteoblasts given overlap in machinery. If this were the case, loss of estrogen as in menopause, would be expected to downregulate glucose transporters on osteoblasts and glycolysis, thereby reducing osteoblast activity.

Osteoclast differentiation is accompanied with an accelerated and activated metabolic program that generates energy for modulating bone resorption (Taubmann et al., 2020; Da et al., 2021). The metabolic adaptation orchestrated by hypoxia-inducible factor 1-alpha (HIF1α) and C-Myc facilitates osteoclast differentiation (Indo et al., 2013). This is further coordinated *via* an increase in the expression of glucose and glutamine transporters, followed by glycolysis and glutaminolysis (Araujo et al., 2017). Treatment with inhibitors that impede glycolysis, including mammalian target of rapamycin (mTOR) and adenosine monophosphate (AMP) activated protein kinase (AMPK), induces osteoclastogenesis inhibition (Li et al., 2018). Taubmann et al. (2020) characterized bone resorbing osteoclasts with increased glycolysis and lactate production *in vitro*. However, the *in vivo* outcome of these events remains poorly understood. The blockage of glycolysis and lactate production interferes with pathological osteoclasts mediated bone loss during OVX as a post-menopausal osteoporosis model (Taubmann et al., 2020). This mechanism enhanced the differentiation and activation of osteoclasts. Despite an increase in bone density in osteoporotic mice, the reduced bone mass was observed in healthy mice upon the blockage of glycolysis (Donat et al., 2021). The reason behind such observation might be due to the adverse effect of glycolysis blockage on new bone forming osteoblasts and osteocytes.

#### 3.1.2 Lipid handling

Fatty acids serve as energy dense substrates for oxidative phosphorylation capable of yielding more ATP compared to glucose or glutamine. As such, Adamek and others identified fatty acids yet another substrate capable of supplying energy to bone tissue and isolated cells in 1987 (Adamek et al., 1987). Subsequent studies have further provided support that long chain fatty acids are critical for proper osteoblast function (Kim et al., 2017). In this capacity, several lines of evidence lead credence to the potential of lipid metabolism affecting osteoblast during menopause. Estrogens have been shown shift from lipid storage to its oxidation for ATP generation in other tissues (Luo et al., 2017; Alemany, 2021). Therefore, it is speculated that removing estrogenic signals on osteoblasts would impair oxidation and ATP generation from fatty acid substrates, reducing bone formation. Additionally, while estrogens may not impact lipolysis directly, they have been shown to protect against hepatic steatosis and lower cholesterol acyl-transferase (Tian et al., 2012). Interesting, a similar pathology of lipid droplet accumulation has been demonstrated in osteoblast and osteocytes associated with low bone mass (Rendina-Ruedy and Rosen, 2020). Therefore, it’s feasible that lipid accumulation occurs in bone during menopause and again exerts a negative impact on bone. Estrogen also controls mitochondrial biogenesis and function (Klinge, 2008). Taken together, these data support the premise that estrogen withdrawal results in mitochondrial dysfunction, likely negatively impacting osteoblast function as well. To support this further, only female mice which have impaired osteoblast long chain fatty acid metabolism by generating osteocalcein (OCN)-Cre.Cpt2^fl/fl^ mice manifest severe skeletal impairments (Kim et al., 2017). Seeing as male mice were protected from this phenotype as their osteoblasts were able to compensate for loss of fatty acid metabolism using other substrates, the estrogen in female mice made osteoblasts more dependent on fatty acids (Kim et al., 2017). A recent study done by Kushwaha et al. (2022) has illustrated the crucial role of mitochondrial long chain fatty acid oxidation during osteoclast differentiation and in typical bone resorption. Nevertheless, the role of estrogen and the molecular pathway regulating the fatty acid oxidation in osteoclast differentiation is unknown. Since several studies have led insights about the importance of estrogens in regulating fatty acid oxidation in different tissues including that in osteoblasts (Kim et al., 2017), the potential involvement of estrogen during the intermediary osteoclast metabolism and the inherent downstream impact arising due to the estrogen deficiency could be worthy of further study. An additional line of evidence strongly suggests that osteoblast bioenergetics are modified during estrogen withdrawal involves to Wnt-LRP5 signaling pathway. In this capacity, estrogen has been shown to enhance Wnt signaling, particularly during loading or mechanical stimulation. Interestingly, Frey et al. (2015) have demonstrated that Wnt-LRP5 signaling regulates fatty acid metabolism in osteoblasts and is consequently responsible for the profound skeletal phenotypes associated in these transgenic mice. Again, given these data it’s likely that estrogen also increases osteoblast fatty acid utilization to support cellular function and bone formation. Therefore, during menopause or loss of estrogen, such metabolic pathways are expected to be impaired, and likely contributing to bone loss.

#### 3.1.3 Other mechanisms

##### 3.1.3.1 Adipose tissue

As described previously, adipose tissue redistribution and accumulation occurs during menopause. While we have used these data to describe alterations in systemic metabolism and intracellular lipid handling, the bone-fat connection has been a heavily studied area. Generally, as visceral adipose tissue expands, there is general decrease in BMD. It’s true that increasing adiposity also increases loading or mechanical innervation of the skeleton, which can have a beneficial impact on bone (Gkastaris et al., 2020). However, it is accepted within the field that systemic inflammation and/or glucose intolerance associated with obesity results in bone fragility and increased fracture occurrence (Turcotte et al., 2021). Aging, menopause, obesity with or without diabetes all of which has been shown to be associated with bone loss or bone fragility have been shown to cause bone marrow adipose tissue expansion (Ali et al., 2022). Noteworthy, recent work has demonstrated that in addition to estrogen, FSH also has profound impact on adipose tissue. The late perimenopause period is characterized with rapid bone loss and enhanced visceral adiposity (Thurston et al., 2009). A study performed by Liu et al. reported that reducing FSH signaling using a monoclonal antibody targeted to both the human and mouse FSHβ not only limits subcutaneous and visceral fat but also causes profound beiging, coupled with the acceleration of mitochondrial density and enhanced thermogenesis (Liu et al., 2017). Hence, it could become therapeutic target in the treatment of both postmenopausal osteoporosis and obesity.

Inflammation of adipose tissue rose to scientific prominence in the mid-1990s, shortly after obesity was recognized as an inflammatory disease in a rat model, specifically demonstrating a striking increase in TNFα (Hotamisligil et al., 1993). This has been an area of much scientific inquiry as this fat depot has been identified to be a source of many pro-inflammatory cytokines which can circulate systemically. For example, expansion in visceral adipose tissue is correlated with elevated C-reactive protein (CRP), IL-6, and TNFα. Perhaps even more provocative, a number of single-gene mutations are known to extend lifespan in lower organisms, and similar lifespan extension is observed even if the mutations are restricted to adipose (Blüher et al., 2003). These data, therefore, begin to set the precedence that adipose tissue hypertrophy associated with menopause are a major contributor to the inflammation process. This hypertrophy leads to adipocyte ‘crowning’ which refers to the crown-like structure observed where macrophages surround dead or dying adipocytes (Murano et al., 2008). These adipose tissue macrophages (ATMs) secret high levels of proinflammatory cytokines and demonstrate inflammasome activation (Cai et al., 2022). While ATMs provide a significant source of proinflammatory cytokines, *in vitro* studies also demonstrate that adipocytes themselves can produce such cytokines as well, directly contributing to systemic inflammation. Such pro-inflammatory cytokines have been associated with decreased osteoblast activity but have not been studied within the context of modulating cellular metabolism.

In addition to the classic white adipose depots above, bone marrow adipocytes may also provide clues between how adipocytes can interact with osteoblasts during estrogen withdrawal. Bone marrow adipocytes found interspersed throughout the marrow compartment are identified based on the morphology of their defining unilocular lipid droplet, and collectively are often referred to as bone marrow adipose tissue (BMAT) (Horowitz et al., 2017). Remarkably, BMAT constitutes over 10% of total body fat in lean and healthy adults (Attané et al., 2020)representing 50%–70% of the total bone marrow cavity (Hindorf et al., 2010; Zhong et al., 2020; Nandy, 2021). Initially thought to be inert space-fillers, recent studies have confirmed the contribution of BMAT in a varying range of physiological as well as clinical aspects (Li et al., 2019). Once identified, observational studies began to report that BMAT is often inversely associated with bone mineral density (BMD). For example, this relationship has consistently been demonstrated during aging (Shen et al., 2014), post-menopausal osteoporosis (Shen et al., 2014), anorexia nervosa (Bredella et al., 2009), as well as during obesity (Elia, 2012; Rinonapoli et al., 2021; Ali et al., 2022). In these scenarios, patients often present with an increase in BMAT along with a decrease in bone mass or BMD (93). Indeed, bone marrow adipocytes have been shown to regulate hematopoietic activity (Cuminetti and Arranz, 2019) and possibly regulate systemic metabolism, however, much more research is needed to fully understand the precise function of these unique cells and the mechanism which coordinates bone marrow adipocyte-bone cell communication axis. Given the intimate proximity of bone marrow adipocytes to osteoblasts and osteoclasts, along with their precursors, it stands to reason that BMAT can directly influence bone mass by secreting factors to regulate skeletal homeostasis. In fact, we have been able to demonstrate that factors secreted from bone marrow adipocytes can be transferred to osteoblasts. Our lab has recently demonstrated that upon lipolytic stimulation of PTH, bone marrow adipocytes are able to transfer free fatty acids to osteoblasts (Maridas et al., 2019). During menopause, BMAT expands as bone formation wanes. Thus, it is possible that fatty acid substrates once destined to fuel bone formation *via* osteoblast utilization, are now unavailable as they accumulate in the marrow adipocyte. This hypothesis has been somewhat corroborated as sophisticated studies by Li et al. (2022) who demonstrated in other models of bone loss, bone marrow adipocyte lipolysis is critical for bone formation.

## 4 Microbiome

Not surprisingly, it’s been suggested that gut microflora and their structural diversity, phenotypic and genotypic composition are responsive in maintaining physiological bone metabolism. Accumulating evidence suggests the indispensable role of gut microbiome in post-menopausal osteoporosis development. For example, germ free mice do not exhibit any striking changes in femoral trabecular parameters compared to conventional mice (Sjögren et al., 2012). Even so, both conventional mice and microbial colonized germ-free mice manifest higher pro-inflammatory cytokines and impaired bone features due to the lack of estrogen. It is reported in murine model, owing to the sex steroid deficiency, there is an increased gut permeability that offers cross-link between the gut-bone axis (Shieh et al., 2020). Besides, it also expands pro-inflammatory Th17 cells, up regulating osteoclastogenic cytokines. To the contrary, germ-free mice that lack sex steroid hormone failed to increase osteoclastogenic cytokines production resulting in protection from trabecular bone loss (Alam et al., 2016). This supports the finding that gut microbiome is crucial in sex-steroid deficiency triggered bone loss.

Estrogen modulates the intestinal microbiome in an interplay tailored to enhance the proliferation of beneficial bugs (Chen and Madak-Erdogan, 2016) Estrogen activates regulatory T-cells (Tregs), that prevents osteoclastogenesis and promote bone mass. *Clostridi* *a* are well known producers of SCFAs, including butyrate. Butyrate maintains gut epithelial barrier, demonstrate immunomodulatory characteristics, and thus activates regulatory T cells (Furusawa et al., 2013). Another study done by Attarshi et al. reported that human *Clostridia* induce Tregs activity and contribute to systemic skeletal homeostasis (Atarashi et al., 2013). Lucas et al. performed a study comparing mice fed with short chain fatty acids (SCFAs) or high fiber diet and found a considerable increase in bone mass preventing postmenopausal and inflammatory bone loss (Lucas et al., 2018). Moreover, the SCFAs propionate (C3) and butyrate inhibit osteoclast differentiation and limits bone resorption *in vitro* and *in vivo* (Wallimann et al., 2021). Even more significantly, these two SCFAs incite osteoclast metabolism resulting in increased glycolysis at the expense of oxidative phosphorylation and thus downregulates osteoclast genes such as TRAF6 (TNF Receptor Associated Factor 6) and NFATc1 (Nuclear Factor of Activated T Cells 1) (Lucas et al., 2018).

Estrobolome (gene repertoire of the gut microbiota) metabolize estrogens *via* secretion of β-glucuronidase (Baker et al., 2017). The dysbiosis of intestinal microbiota impairs deconjugation of estrogen resulting in a decrease of circulating estrogen and disrupting the homeostasis. It is crucially worthy to note that loss of estrogen is associated with unfavorable alterations in the gut microflora, weakening gut epithelial barrier’s integrity, thereby increasing the intestinal permeability with reduced calcium absorption and enhanced inflammatory response (Baker et al., 2017). The therapeutic approaches to exploit the endocrine landscape of estrogen’s actions while preserving the integrity of the gut microbiome could offer a potential therapy to prevent estrogen depletion-induced bone loss. Genetic history is also one of the key drivers in maintaining basal bone mass as well as the specific distribution of intestinal antigen presenting cells (APCs) with various functions. In psost-menopausal osteoporosis, the bone resorption activity is closely adhered to genetic background. Various studies with estrogen deficient mice have reported the striking variation in bone loss among diverse mouse strains (Iwaniec et al., 2006). Intestinal APCs, dendritic cells (DCs), macrophages and ovariectomy enhanced T cell production TNF-α augments RANKL-induced osteoclastogenesis and stimulates bone loss (Cenci et al., 2000; Pacifici, 2012). It’s not surprising that the structural composition of intestinal microbiome varies based on the host genetic background and thereby can shape the functional activity of the host immune system and thus indirectly contributes to regulating bone loss in PMO. To explain the crucial interconnection between gut microbiome and energy metabolism in the human body, it seeks further comprehensive investigation.

## 5 Conclusion

Type I osteoporosis being the most prevalent form of low bone mass affecting one in 10 women over the age of 60. Therefore, it is pertinent to understand the molecular mechanisms leading to this disease so that we can develop novel targeted strategies to prevent fracture. This review has focused on the multiple factors that contribute to post-menopausal osteoporosis. while underscoring metabolic homeostasis at a global and cellular level. Hormonal changes associated with this stage of women life has been found to be the major contributor to the bone loss. However, since menopause happens after 50 the age-related consequences can also play an important role in this type of osteoporosis. Menopause is associated with loss of estrogen that further leads to a sequential functional change thereby affecting bone. It is very intriguing that loss of one hormone can affect so many bodily functions and so many different tissues and organs. Loss of this hormone can alter the entire bone dynamics by both increasing resorption and decreasing formation. Estrogen not only directly affects the differentiation of the precursor cells more towards active osteoclasts and less towards bone forming osteoblasts but also can affect their cellular energetics. Increased adiposity and inflammation post menopause can also indirectly lead to loss of bone mass. Post-menopausal osteoporosis is an impeccable example of how altered endocrine function can affect both systemic and tissue specific functions as well as cross talk between different organs to lead to a complex multifactorial disease. Interestingly, gut microbial population regulated by estrogen and genetic background of the individual can also contribute to development of post-menopausal osteoporosis. Although, estrogen has been shown to directly affect osteoclastogenesis and gene expression for resorption, the metabolism related mechanisms are needed to be studied further to make a conclusion. There are few studies of how estrogen can modulate bone homeostasis by regulating metabolism of osteoblasts and osteoclasts. It will be very interesting to study these aspects, to compare metabolism of bone cells in young versus menopausal organisms understand how cellular bioenergetics is altered in bone cells during the ageing process along with gonadal deficiency.
